# Vaccination, time lost from work, and COVID-19 infections: a Canadian healthcare worker retrospective cohort study

**DOI:** 10.3389/fpubh.2023.1214093

**Published:** 2023-08-07

**Authors:** Arnold I. Okpani, Karen Lockhart, Jennifer M. Grant, Stephen Barker, Jocelyn A. Srigley, Annalee Yassi

**Affiliations:** ^1^School of Population and Public Health, University of British Columbia, Vancouver, BC, Canada; ^2^Medical Practitioners Occupational Safety and Health, Vancouver Coastal Health, Vancouver, BC, Canada; ^3^Department of Pathology and Laboratory Medicine, Faculty of Medicine, University of British Columbia, Vancouver, BC, Canada; ^4^BC Children’s and Women’s Hospital Research Institute, Vancouver, BC, Canada

**Keywords:** COVID-19, healthcare workers, vaccination, occupational health, side effects

## Abstract

The COVID-19 pandemic highlighted hurdles for healthcare delivery and personnel globally. Vaccination has been an important tool for preventing severe illness and death in healthcare workers (HCWs) as well as the public at large. However, vaccination has resulted in some HCWs requiring time off work post-vaccination to recover from adverse events. We aimed to understand which HCWs needed to take time off work post-vaccination, for which vaccine types and sequence, and how post-vaccination absence impacted uptake of booster doses in a cohort of 26,267 Canadian HCWs. By March 31, 2022, more than 98% had received at least two doses of the approved COVID-19 vaccines, following a two-dose mandate. We found that recent vaccination and longer intervals between doses were associated with significantly higher odds of time-loss, whereas being a medical resident and receiving the BNT162b2 vaccine were associated with lower odds. A history of lab-confirmed SARS-CoV-2 infection was associated with lower odds of receiving a booster dose compared with no documented infection, aOR 0.61 (95% CI: 0.55, 0.68). Similarly, taking sick time following the first or second dose was associated with lower odds of receiving a booster dose, aOR 0.83 (95% CI: 0.75, 0.90). As SARS-CoV-2 becomes endemic, the number and timing of additional doses for HCWs requires consideration of prevention of illness as well as service disruption from post-vaccination time-loss. Care should be taken to ensure adequate staffing if many HCWs are being vaccinated, especially for coverage for those who are more likely to need time off to recover.

## 1. Introduction

Globally, the COVID-19 pandemic has caused disruptions to healthcare delivery (1–3), with significant pressures on the healthcare workforce. Potential occupational infectious exposures (4–6), heavy patient burdens, mental health stresses, stigmatization, and concerns about family transmission (7–11) have all contributed to health care worker (HCW) stress. One factor that has become much more prominent is the problem of staff shortages (12, 13). When vaccines became available, HCWs were prioritized for vaccination to decrease their risk of infection (14), reduce transmission in healthcare and ensure maximal staffing, given the primary role that vaccination plays in infection prevention, especially in HCWs (15). Since then it has become apparent that vaccines, though excellent at reducing risk of hospitalization and severe disease (16), are not as effective at stopping infection and transmission of recent variants of SARS-CoV-2 (17).

Booster doses have been recommended to HCWs with the expectation that it will maintain the benefits of vaccination and reduce absenteeism. Some studies have shown booster doses inducing a significant and sustained increase in anti-SP IgG titers (18) while others have found a more short-lived impact (19). Nonetheless, there is a growing body of evidence on the side effects of COVID-19 vaccines and resulting time off work (20–22), which should not be ignored. The extent of post-vaccination absenteeism is, therefore, an important issue to explore in order to understand the characteristics that are associated with time lost from work, within occupational groups and demographics as well as in respect to vaccination type and timing. A good understanding of these factors is important to help policy makers as well as individual HCWs and their supervisors to take the potential side effects into consideration in decision-making, and minimize service disruptions that may be associated with vaccination.

The objective of this study was to characterize and assess the occurrence of post-vaccination absenteeism among HCWs in the Vancouver Coastal Health (VCH) authority of British Columbia (B.C.), Canada after two doses of the vaccine as well as after booster doses and to investigate any association between previous infection and adverse events following immunization (AEFI).

## 2. Methods

### 2.1. Study context and data

Vancouver Coastal Health is one of seven health authorities in B.C. providing health care for approximately 1.25 million patients. In B.C. HCWs were mandated to receive 2 doses of vaccine against COVID-19 in Fall of 2021. Long-term care workers (LTC) were to be vaccinated before October 12, 2021 (23), and those working in acute care and other publicly-funded healthcare facilities were to be vaccinated by October 26, 2021 (24). This study used data on HCW SARS-COV-2 tests and vaccination records that are available in the provincial occupational health database, which captures data regarding vaccination and COVID-19 tests. These included staff providing all laboratory, community, hospital, and LTC services. Data on medical staff (physicians, midwives, nurse practitioners, and dentists) were not included as they work mostly as independent contractors, rather than as employees of the health authorities in the province. Non-medical contractors (e.g., cleaning and food service staff) are also not included in our cohort. Data were extracted on VCH employees from December 15, 2020, when vaccines were initially provided to HCWs, until March 31, 2022. Only active employees were included, defined as those with an active position at the end of the observation period on March 31, 2022, and working for at least 3 months during the observation period. All workers included in our cohort were employees of the health authority; the vast majority were unionized, and all were entitled to paid sick time if off work for illness.

Data were extracted on HCW age, sex (male, female, unspecified), job category (nurses, administration, allied health, licensed practical nurses [LPN]/care aides, support staff, medical residents, and other), daily shift data including productive shifts and sick leave. Details on COVID-19 vaccination (date, dose number, type - BNT162b2, MRNA-1273, other) and dates of all SARS-CoV-2 test results were also obtained. The reason for sick leave was not available. All personally identifiable information was removed prior to data extraction.

### 2.2. Statistical analysis

A descriptive analysis of all active HCWs within VCH was conducted. This analysis included the number and proportion of workers that fell within different categories: age group, gender, job category, COVID test history, and the number of vaccines received. Age group and number of vaccine doses were calculated as of March 31, 2022. In addition, categorized counts were calculated for the proportions of HCWs that used at least 1 day of sick leave within 3 days of any COVID-19 vaccine dose. Next, a multivariable logistic regression was conducted to estimate the association between the outcome of a worker-day including sick leave with the worker day being within 3 days of a vaccine dose, the type of vaccine provided, and the dose number. Multivariable logistic regression was used to estimate the association between the outcome of sick leave in the 3 days following vaccination with the vaccine type sequence, timing of vaccine doses and job category as well as to estimate the association of the outcome of receiving a booster vaccine with whether the worker had previously tested positive or had taken sick days in the 3 days following the first two doses. For multivariable logistic regression, the odds ratio (OR) and 95% confidence interval (CI) were estimated, with *p*-values ≤0.05 considered statistically significant. As a sensitivity analysis, we set the outcome of number of sick days taken in the 3-days following vaccination, per worker, grouped by vaccine dose as a continuous variable.

Fourteen worker-days following a positive SARS-CoV-2 test result were excluded from analysis, as a recent positive test for SARS-CoV-2 has an extremely strong correlation with subsequent sick days off, consistent with public health isolation requirements even if the HCW was not particularly ill; there was also an extremely low number of vaccinations provided during this period for a worker.

Statistical analyzes were conducted using R statistical software, version 4.2.3.

This study was approved by the Behavioral Ethics Review Board at the University of British Columbia, certificate number H21-01138.

## 3. Results

Data on 26,267 HCWs were included in the analysis. A third of the cohort were under age 35, and 81% were females. At the end of the period of interest in this study, more than 21,000 HCWs (80.3%) had received at least three doses of the approved vaccines. Table 1 presents the characteristics of the study population.

**Table 1:** Cohort of healthcare workers in Vancouver Coastal Health with at least 3 months of active service between December 15, 2020 and March 31, 2022.

Characteristics	Total		Used ≥ 1 sick day within 3 days of a vaccine (any dose)	
	*N*	%ᵃ	*N*	%ᵇ
All workers	26,267	100.0%	5,832	22.2%
Age group				
Under 35	8,404	32.0%	1,500	17.8%
35 to 44	6,414	24.4%	1,494	23.3%
45 to 54	5,426	20.7%	1,451	26.7%
55 or older	6,023	22.9%	1,387	23.0%
Gender				
Men	4,747	18.1%	954	20.1%
Women	21,164	80.6%	4,839	22.9%
Unspecified	356	1.4%	39	11.0%
Job categoryᶜ				
Nurses	8,125	30.9%	1,704	21.0%
Administration	4,987	19.0%	1,076	21.6%
Allied health	5,225	19.9%	1,280	24.5%
LPN/care aide	5,722	21.8%	1,468	25.7%
Support staff	926	3.5%	215	23.2%
Resident	1,400	5.3%	74	5.3%
Other	705	2.7%	14	21.0%
COVID test history (prior to March 31, 2022)				
Positive at least once	2,703	10.3%	672	24.9%
Never tested or only tested negative	23,564	89.7%	5,160	21.9%
Vaccination status (on March 31, 2022)				
3+ doses	21,090	80.3%	4,808	22.8%
2 doses	4,807	18.3%	1,006	20.9%
1 dose	92	0.4%	18	19.6%
Unvaccinated/no record	278	1.1%	-	-

ᵃPercent is the proportion of all workers. ᵇPercent is the proportion of workers with at least 1 sick day within the classification group. ᶜA single person can have positions in multiple job categories and can be counted more than once. Medical staff (physicians, nurse practitioners, dentists, and midwives) are excluded as sick days are not reported. LPN = licensed practical nurse.

### 3.1. Vaccination, vaccine type and sick days

**Figure 1:**
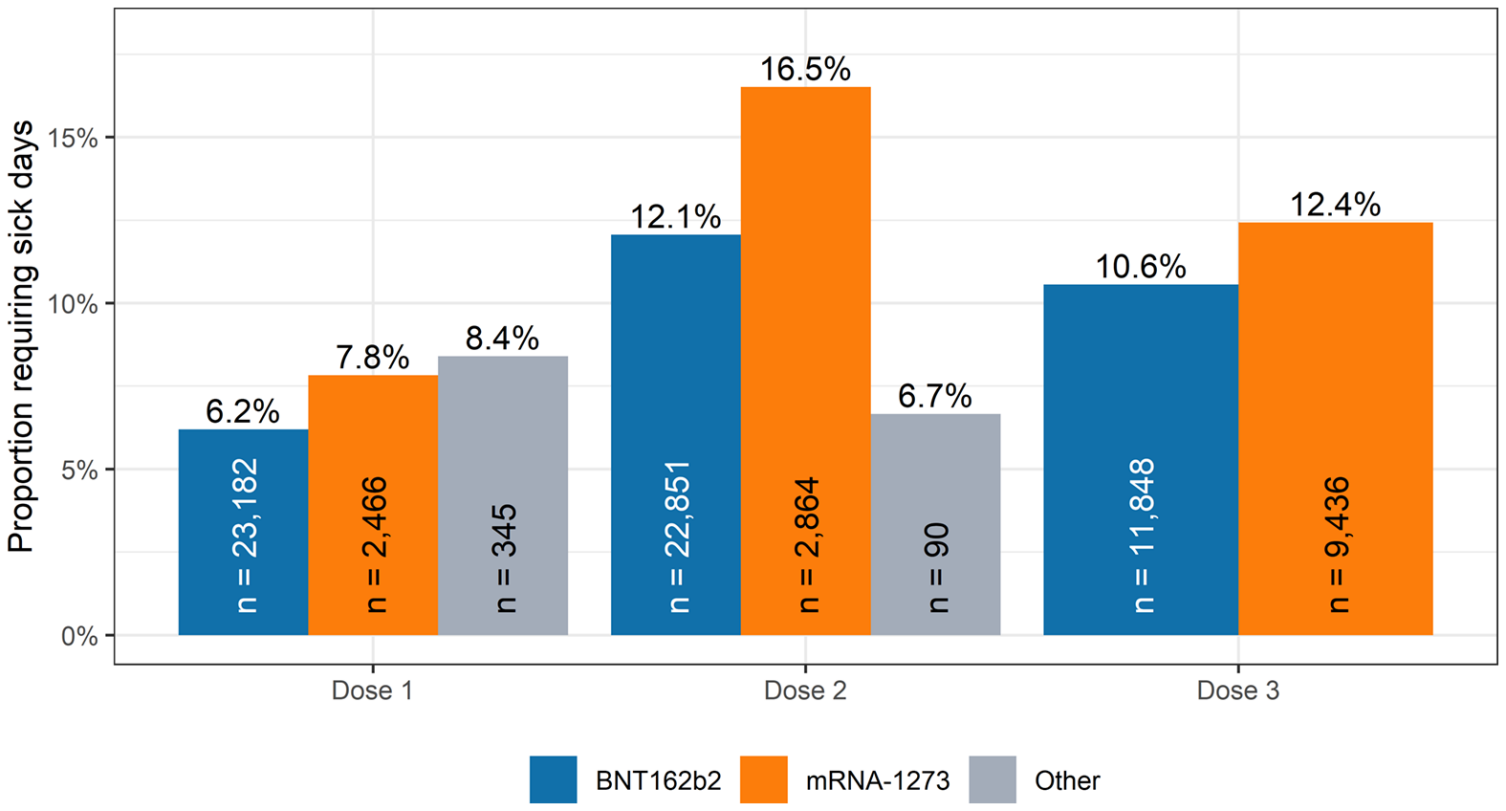
Proportion of health care workers requiring sick days within 3 days post-vaccination. Vancouver Coastal Health December 2020–March 2022.

Figure 1 shows the proportion of HCWs who took a sick day following vaccination. More HCWs were vaccinated with BNT162b2 in each dose round. The proportion of HCWs requiring sick days were lower in each round for BNT162b2 recipients when compared with mRNA-1273 recipients. Table 2 shows the result of multivariable logistic regression analysis for the association between recent vaccination, vaccine type and dose with sick days. The odds of taking a sick day (regardless of cause) were significantly higher for mRNA-1273 and other approved vaccines (including ChAdOx1 and Ad26.COV2.S) compared to BNT162b2, aOR 1.50 (95% CI: 1.43,1.57) and 2.02 (95% CI: 1.54,2.64), respectively. Similarly, second and third dose vaccinations were associated with significantly more sick time compared to the first dose, aOR 1.98 (95% CI: 1.88,2.09) and 1.69 (95% CI: 1.60,1.79), respectively. When we set the outcome as a continuous variable, we found that the average number of sick days taken in the 3-day period after vaccination is consistent with this result, showing the second dose associated with the largest mean sick days per worker and the first dose with the least.

**Table 2:** Multivariable adjusted odds ratio of all-cause sick leave for any vaccine dose among health care workers in Vancouver Coastal Health (December 2020–March 2022).

Variable	Total worker days	All-cause sick time worker days (% of total)	Adjusted odds ratio of all-cause sick time (95% CI)ᵃ
Recent vaccination			
Not vaccinated within 3 days prior	18,651,917	1,042,508 (5.6%)	(ref.)
Vaccinated within 3 days prior (any COVID-19 vaccine)	102,387	9,738 (9.5%)	1.80 (1.76, 1.84)
Vaccine type			
BNT162b2	80,945	7,128 (8.8%)	(ref.)
mRNA-1273	20,912	2,538 (12.1%)	1.50 (1.43, 1.57)
Other	437	64 (14.6%)	2.02 (1.54, 2.64)
Vaccine dose			
1st dose	35,132	2,263 (6.4%)	(ref.)
2nd dose	35,781	4,268 (11.9%)	1.98 (1.88, 2.09)
3rd dose	31,461	3,203 (10.2%)	1.69 (1.60, 1.79)

^a^Adjusted for age, sex, and job category. CI, confidence interval.

Table 3 shows the relationship between vaccine sequence, interval between doses, job category and the outcome of sick time following each dose. The odds of taking a sick day following first dose vaccination was significantly higher for recipients of mRNA-1273 compared to BNT162b2; 1.31 (95% CI: 1.07,1.61). For second dose vaccinations, whereas those who took mRNA-1273 following a first dose with BNT162b2 did not have significant difference in odds of sick days compare to those who took BNT162b2 for both doses, the odds of sick days for those who took mRNA-1273 for both doses was significantly higher, aOR 1.55 (95% CI: 1.38,1.74). By the third dose (first booster shot), only HCWs who took mRNA-1273 for the first time following two earlier doses of BNT162b2 required more sick time compared to those who took BNT162b2 for all three doses.

**Table 3:** Multivariable adjusted odds ratio of sick leave by vaccination sequence, interval and job category among health care workers in Vancouver Coastal Health (December 2020 – March 2022).

Variable	Sick time after dose 1 (95% CI)	Sick time after dose 2 (95% CI)	Sick time after dose 3 (95% CI)
Vaccine type and sequence^a^			
BNT162b2	(ref.)	-	-
mRNA-1273	1.31 (1.07, 1.61)	-	-
BNT162b2 x2	-	(ref.)	-
mRNA-1273 ×2	-	1.55 (1.38, 1.74)	-
BNT162b2, mRNA-1273	-	1.27 (0.97, 1.66)	-
BNT162b2 x3	-	-	(ref.)
BNT162b2 x2, mRNA-1273	-	-	1.30 (1.19, 1.42)
mRNA-1273 ×3	-	-	1.02 (0.84, 1.23)
mRNA-1273 ×2, BNT162b2	-	-	1.16 (0.82, 1.65)
Other vaccine/sequence	0.85 (0.42, 1.75)	0.68 (0.48, 0.96)	0.79 (0.58, 1.06)
Time interval between doses^a^			
Less than 90 days after first dose	-	(ref.)	-
90+ days after first dose	-	1.10 (1.02, 1.19)	-
Less than 180 days after second dose	-	-	(ref.)
180+ days after second dose	-	-	1.25 (1.02, 1.55)
Job category^b^			
Nurses	(ref.)	(ref.)	(ref.)
Administration	1.14 (0.99, 1.33)	1.17 (1.05, 1.31)	1.16 (1.02, 1.32)
Allied health	1.04 (0.90, 1.21)	1.30 (1.17, 1.45)	1.40 (1.24, 1.58)
LPN/care aide	1.35 (1.18, 1.55)	1.31 (1.18, 1.45)	1.72 (1.53, 1.93)
Support staff	1.31 (1.00, 1.73)	1.35 (1.10, 1.67)	1.59 (1.24, 2.04)
Resident	0.42 (0.29, 0.62)	0.20 (0.14, 0.29)	0.30 (0.21, 0.41)
Other^c^	0.10 (0.03, 0.30)	0.12 (0.06, 0.25)	0.20 (0.10, 0.39)

^a^Adjusted for age, gender, job category. ^b^Adjusted for age, gender, vaccine type, time interval between doses. ^c^Other: Student or volunteer. CI, confidence interval.

### 3.2. Vaccination interval, job category, age and sick time

Getting the second dose vaccination 90 or more days after the first dose was associated with significantly higher odds of requiring sick time compared to an interval of less than 90 days, aOR 1.10 (95% CI: 1.02, 1.19). Similarly, the odds of taking sick time were significantly higher for a dosing interval of 180 days or more, compared to less than 180 days between the second dose and the first booster dose, aOR 1.25 (95% CI: 1.02, 1.55).

Administrative staff, allied HCWs, care aides, and support staff had higher odds of taking sick time following vaccination compared to nurses, regardless of the dose. Resident doctors had significantly lower odds of taking sick time after vaccination compared to nurses, regardless of dose.

### 3.3. History of COVID-19, post-vaccination sick day and uptake of booster dose

A history of lab confirmed SARS-CoV-2 infection prior to March 31^st^, 2022, was associated with lower odds of receiving a booster dose compared with no documented infection, aOR 0.61 (95% CI: 0.55, 0.68). Similarly, taking a sick day following the first or second dose was associated with lower odds of receiving a booster dose, aOR 0.83 (95% CI: 0.75, 0.90), though the effect was considerably less than those who had a history of COVID-19 infection.

## 4. Discussion

This study of a large cohort of Canadian HCWs, in which more than 98% had received two or more doses of approved COVID-19 vaccines, found that the odds of taking sick time was significantly higher among HCWs who took a COVID-19 vaccine in the preceding 3 days than for those who did not. The largest impact of vaccination on absence from work was seen following the second vaccine dose. The third vaccine dose (first booster) was associated with more absences than the first but less than the second dose. The mRNA-1273 vaccine was associated with more absenteeism compared to BNT162b2, which was the most frequently administered vaccine in the cohort.

Longer intervals between vaccine doses and occupation were both important predictors of post-vaccination absence. Of particular note, resident doctors were the least likely to be absent of all employee groups. The finding that medical residents were among the least likely to report post-vaccination absence supports previous reports (25, 26) which indicated that resident doctors were particularly more likely to continue working when ill (27). Jena et al., in a survey of residents, stated that they were frequently under pressure from colleagues to continue working while ill or due to inadequate personnel to cover for them. In that study, 51% of the residents surveyed stated that they worked with flu-like symptoms at least once in the preceding year (25). Presenteeism (working while ill) not only compromises patient safety, but also negatively impacts HCW productivity and mental well-being (28–30). Likewise, nurses were less likely to take time off in the 3 days following vaccination compared to most other HCWs except for residents. Other investigators have reported that acceptability of AEFI which may vary by occupational group could be an important factor in this regard (31). Other factors may relate to availability of sick time benefits. Indeed unionization, pay, and job security are known to influence illness-related absence from work (32). These factors, and variation by occupation noted in this study, merit further investigation so as to better understand the risks of presenteeism or pressure to work if wards are short-staffed.

In examining the association between both previous SARS-CoV-2 infection and post-vaccination absence on one hand, and the uptake of the recommended vaccine booster dose on the other, it was found that both factors were associated with a lower uptake of the booster dose. Past infection was a stronger predictor, with 39% lower odds of receiving a booster dose compared to those without a prior positive COVID-19 test.

Given the very high uptake of COVID-19 vaccination in this cohort of HCWs and the possibility that public health authorities may recommend periodic booster doses (33), the impact that vaccination had on time lost from work merits consideration. Whereas previous studies in this HCW population by our team demonstrated that vaccines were effective early in the pandemic in protecting HCWs against COVID-19 infection (14, 34) – with its consequent time loss due to isolation requirement or due to severe illness – this present study highlights an area of attention, especially for any future vaccination calls that may lead to many HCWs seeking vaccination within a short period. Canada initiated its COVID-19 vaccination campaign on December 14, 2020 to first administer doses to priority groups (i.e., those at high risk of severe illness and death from COVID-19 and those who were likely to be exposed to the virus: residents and staff of congregate living settings, frontline HCWs, adults in Indigenous communities, adults of advanced age) (35). The first and second doses were mandatory for all HCWs in British Columbia but different provinces had differing rules.

Several other studies also reported that mRNA COVID-19 vaccines have had side effects that have prompted many to limit daily activities or miss work post-vaccination (20, 21, 36). Similar to the results reported here on vaccine types, Cohen et al. found a significant difference in the proportion of those who reported needing to miss work after the second dose, with 49.4% of those who received the higher dose mRNA-1273 vaccine needing to miss work, versus 26.2% of those who received BNT162b2 (37).

The foregoing is not intended to downplay the benefit of HCW COVID-19 vaccination in reducing time loss, which has been reported in other studies, including those by Maltezou et al. and Paladino and colleagues. In addition, the literature has highlighted the importance of vaccination for the whole population, with lower hospital and intensive care patient numbers associated with high vaccine coverage (38). Specifically that study found that vaccination provided a significant protective effect when at least 40% of people are vaccinated, whatever the dose considered (38).

Although the specific reasons for reduced uptake of booster doses among previously infected HCWs merits further investigation, there are numerous reports associating history of past SARS-COV-2 infection with lower likelihood of receiving a booster dose of the vaccine (21, 39–41). Whist our analysis found that a history of infection was associated with 39% lower odds of booster uptake in this cohort, Viskupic and Wiltse reported 49% lower odds among nurses in South Dakota (41). Others have suggested that a potential explanation for this reluctance is greater concern among people who had had COVID-19 infections about experiencing side-effects following mRNA vaccination related to already having been infected (42, 43).

HCWs in our cohort who had a sick day after their first or second doses were less likely to get a booster dose than their co-workers who did not require sick time, which echoes what other investigators have found (44, 45). Chrissian et al. reported that HCWs who missed work post-vaccination were far more likely to cite concerns over side effects as the reason for their reluctance to receive booster doses than any other reason (21). The phrase “post-positive reluctance” was coined to describe the phenomenon (44). Vaccine side effects such as fever, chills, fatigue, and muscle pain may be signs of reactogenicity and that the vaccine is “working,” however, it is imperative that HCWs consider these effects in planning the timing and type of vaccination and that their managers consider the likelihood of these side effects interfering with the ability to work.

### 4.1. Strengths and limitations

This study used data from a robust database of HCW SARS-CoV-2 infection and vaccination with acquisition from the start of the pandemic. Using administrative data, captured by the health authority, ensured that all data were systematically collected, as opposed to questionnaire data, which may rely on volunteer bias and/or recall of individual HCWs. However, the use of secondary administrative data limits the ability to probe some relationships. For example, while HCW sick days were tracked by payroll and therefore accuracy was not an issue, the cause of illness/sick time is not tracked by the employer. Therefore, it is possible that some episodes of illness coincided with, rather than resulted from, vaccination. Nonetheless, if this was the case, this would not be expected to differ systematically between HCWs with recent vaccination and those without. In addition, the proliferation of home rapid antigen test kits for SARS-COV-2 infection in Canada, coinciding with the arrival of the Omicron subvariant of the virus, meant that accurate tracking of HCW infections beyond March 2022 was not possible.

## 5. Conclusion

A principal goal of vaccination is to ensure that healthcare workers have the best protection possible, not just for their own sake but for the safety of their families, colleagues, and patients. Particularly in the early stages of the pandemic, vaccination was widely shown to have reduced severe disease when infection occurred, thus helping to ensure that HCWs are available sooner to provide much needed service. The benefits of vaccination notwithstanding, service disruption from post-vaccination time loss can be minimized if care is taken to ensure adequate staffing in periods when many HCWs are being vaccinated, especially for coverage of those who are more likely to need time off to recover.

## Data availability statement

The data analyzed in this study is subject to the following licenses/restrictions: Data for this study was compiled from anonymized payroll/workplace systems. It can be made available by the authors if requested. Requests to access these datasets should be directed to stephen.barker@ubc.ca.

## Ethics statement

The studies involving human participants were reviewed and approved by University of British Columbia Behavioral Ethics Review Board. Written informed consent for participation was not required for this study in accordance with the national legislation and the institutional requirements.

## Author contributions

AO, JG, JS, and AY contributed to conception and design of the study. SB organized the database and performed the statistical analysis. AO, KL, and AY wrote the first draft of the manuscript. All authors contributed to manuscript revision, as well as read and approved the submitted version.

## Conflict of interest

The authors declare that the research was conducted in the absence of any commercial or financial relationships that could be construed as a potential conflict of interest.

## Publisher’s note

All claims expressed in this article are solely those of the authors and do not necessarily represent those of their affiliated organizations, or those of the publisher, the editors and the reviewers. Any product that may be evaluated in this article, or claim that may be made by its manufacturer, is not guaranteed or endorsed by the publisher.
